# Structural and Chemical Evolutions of Li/Electrolyte Interfaces in Li‐Metal Batteries: Tracing Compositional Changes of Electrolytes under Practical Conditions

**DOI:** 10.1002/advs.202204812

**Published:** 2022-11-18

**Authors:** Youngseong Jo, Dahee Jin, Minhong Lim, Hyuntae Lee, Hyeongguk An, Jiyeon Seo, Gunyoung Kim, Xiaodi Ren, Yong Min Lee, Hongkyung Lee

**Affiliations:** ^1^ Department of Energy Science and Engineering Daegu Gyeongbuk Institute of Science and Technology (DGIST) Daegu 42988 Republic of Korea; ^2^ Department of Materials Science and Engineering University of Science and Technology of China Hefei 230026 China; ^3^ Energy Science and Engineering Research Center Daegu Gyeongbuk Institute of Science and Technology (DGIST) Daegu 42988 Republic of Korea

**Keywords:** electrolyte composition, lean electrolyte, Li/electrolyte interfaces, Li‐metal batteries, reacted Li layer, XPS analysis

## Abstract

Despite the promises in high‐energy‐density batteries, Li‐metal anodes (LMAs) have suffered from extensive electrolyte decomposition and unlimited volume expansion owing to thick, porous layer buildup during cycling. It mainly originates from a ceaseless reiteration of the formation and collapse of solid‐electrolyte interphase (SEI). This study reveals the structural and chemical evolutions of the reacted Li layer after different cycles and investigates its detrimental effects on the cycling stability under practical conditions. Instead of the immediately deactivated top surface of the reacted Li layer, the chemical nature underneath the reacted Li layer can be an important indicator of the electrolyte compositional changes. It is found that cycling of LMAs with a lean electrolyte (≈3 g Ah^−1^) causes fast depletion of salt anions, leading to the dynamic evolution of the reacted Li layer structure and composition. Increasing the salt‐solvent complex while reducing the non‐solvating diluent retards the rate of depletion in a localized high‐concentration electrolyte, thereby demonstrating prolonged cycling of Li||NMC622 cells without compromising the Li Coulombic efficiencies and high‐voltage stability.

## Graphical abstract

Looking underneath the reacted Li layer reveals its structural and chemical evolutions. Contrary to the flooded electrolyte, the reacted Li layer developed at lean electrolyte suffers from incremental deterioration and engenders the cycling history as an important indicator of electrolyte compositional changes, delivering a clue for electrolyte design principle for stable cycling of Li‐metal batteries under stringent conditions.

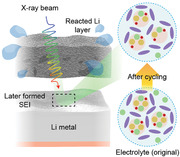

## 1 Introduction

To propel energy density beyond today's lithium (Li)‐ion batteries (LIBs), Li metal anode (LMA) is considered a promising candidate because of its ultrahigh theoretical capacity (3860 mAh g^−1^) and lowest redox potential (–3.04 V vs SHE).^[^ ^1^, ^2^, ^3^, ^4^ ^]^ Instead of a conventional graphite anode, direct implementation of LMA into the LIBs promises a dramatic increase in specific energy (≈500 Wh kg^−1^), which surpasses the maximum limit of the state‐of‐the‐art LIBs.^[^ ^5^, ^6^, ^7^ ^]^ Nonetheless, LMAs have long suffered from deep‐rooted challenges related to the high reactivity with electrolytes, and an inferior solid‐electrolyte interphase (SEI) immediately formed by electrolyte decomposition.^[^ ^8^, ^9^, ^10^ ^]^ Poor SEI quality causes low Coulombic efficiencies (CEs), and irregular Li dendrite growth, leading to electrolyte depletion and high safety risk after prolonged cycling.^[^ ^11^, ^12^, ^13^, ^14^, ^15^, ^16^ ^]^ Therefore, building a highly stable SEI using advanced electrolyte solutions is crucial to achieving the safe and stable cycling of Li metal batteries (LMBs).

Building an SEI at the initial stage passivates the electrode surface at a large negative potential and prevents further electrolyte decomposition during prolonged cycling.^[^ ^3^, ^17^, ^18^, ^19^ ^]^ In contrast to intercalation‐based graphite anodes, the SEI formed at the LMA surface cannot tolerate large volume changes, ultimately leading to a structural collapse during the Li plating/stripping cycles.^[^ ^3^, ^20^, ^21^ ^]^ Such passivation failure allows electrolyte penetration and excessive accumulation of electrolyte‐consuming SEI, resulting in a significant buildup of porous, insulating passivation (the reacted Li) layers, which can increase the cell's impedance and eventually terminate its operation.^[^ ^22^, ^23^, ^24^ ^]^ The LMA surface undergoes dynamic evolution through a detrimental series of repetitive SEI collapses and reformations. Given that the chemical nature of the SEI represents the composition of the electrolyte near the LMA surface,^[^ ^25^, ^26^ ^]^ an in‐depth understanding of SEI dynamics is essential for establishing LMB electrolyte design principles in terms of components and compositions.

Apart from corrosive carbonates, ethers could be a more suitable component for LMAs owing to their excellent cathodic stability.^[^ ^27^, ^28^, ^29^ ^]^ However, the inherently poor oxidation stability restricts their compatibility with high‐voltage (>4 V) cathodes.^[^ ^27^, ^30^, ^31^, ^32^ ^]^ For this purpose, high‐concentration electrolytes (HCEs) excessively increase the salt‐to‐solvent molar ratio, achieving a high voltage tolerance by mitigating the anodic decomposition of free ether solvents.^[^ ^33^, ^34^, ^35^, ^36^, ^37^ ^]^ Moreover, the salt anion‐derived SEI can enrich fluorinated compounds (e.g., LiF), which can further stabilize the SEI at the LMAs, efficiently suppressing Li dendrite growth. Subsequently, the dilution of HCEs with noncoordinating hydrofluoroethers (HFEs) has been suggested to overcome the drawbacks of HCEs, such as high viscosity, poor wettability, and high cost.^[^ ^36^ ^]^ Localized high‐concentration electrolytes (LHCEs) can maintain the unique solvation structure of HCEs while lowering viscosities and improving wettability, further improving the Li CEs (≈99.7%).^[^ ^38^, ^39^, ^40^ ^]^ Indeed, prototypical Li||NMC pouch cells (350 Wh kg^−1^, 2.0 Ah) have demonstrated a long stable cycling under stringent conditions, such as a much lower electrolyte weight to cell capacity (E/C) ratio, and negative (LMA) to positive (NMC cathode) areal capacity (N/P) ratio.^[^ ^12^, ^41^, ^42^ ^]^ Despite advances in electrolytes, LMBs still suffer from extensive growth of the reacted Li layers, which consumes the electrolyte.^[^ ^41^ ^]^ Furthermore, a lean electrolyte undergoes severe compositional changes, which may alter the SEI dynamics during prolonged cycling. In addition, the continuous electrolyte loss upon cycling leads to a poor electrolyte utilization and depletion.^[^ ^43^, ^44^, ^45^ ^]^

Thus, preserving the electrolyte with minimal compositional changes is crucial for improving the persistence of the LMA stabilization. For this purpose, this study is to understand the evolutional behavior of the reacted Li layer during cycling under practical conditions. We revealed the effect of lean electrolytes on the structural and chemical evolution of the reacted Li layer. Given that the reacted Li layer can be developed by the accumulation of SEI formed at the fresh Li surface every cycle, the analysis of the chemistry beneath the reacted Li layer could help speculate that the electrolyte composition evolved as cycling progressed. Moreover, such compositional changes detrimentally affect the electrolyte utilization within the cell, leading to uneven growth of the reacted Li layer and cell failure. Tracing the compositional changes of electrolytes and understanding cycling history can provide insights into the rational tuning of LHCE compositions for practical LMBs. Indeed, we show that the modified LHCE with a tailored diluent ratio demonstrated an improved cycling stability of Li||NMC622 cells with no compromise in the Li CE and high voltage stability.

## 2 Results and Discussion

### 2.1 Understanding the Reacted Li Layer Growth

To investigate the formation and evolution of Li dendrite, it is necessary to study SEI formation and dendrite evolution together with Li CE.^[^ ^14^, ^15^ ^]^ Figure S1 (Supporting Information) shows the common voltage curves for the Li||Cu cell with a baseline carbonate electrolyte (1 M LiPF_6_ in EC/DEC). The CEs suddenly dropped after 40th cycle by gradually increasing the overpotential of the Li plating, which was likely due to the build‐up of the cell impedance triggered by the growth of the reacted Li layer (Figure S1, Supporting Information). Typically, the reacted Li layer is composed of the SEI residues and “dead” Li aggregates. The “dead” Li is the electrically isolated Li piece that is primarily formed during Li stripping owing to random Li dissolution at the Li dendrites.^[^ ^46^, ^47^ ^]^ Such disconnected Li filaments can be deactivated by chemical SEI insulation. The subsequent Li plating mostly occurs beneath the preformed Li/SEI layer. If the newly deposited (“fresh”) Li can partially penetrate the reacted Li layer, it can be embedded in the existing reacted Li layer. At this stage, the “dead” Li and “fresh” Li can coexist in the reacted Li layer. Nonetheless, the “fresh” Li can be easily isolated from the native Li underneath in the following Li stripping and embedded in the reacted Li layer, thereby turning into “dead” Li. Therefore, the top surface of the reacted Li layer becomes deactivated despite being in physical contact with the native LMA underneath.

**Figure 1:**
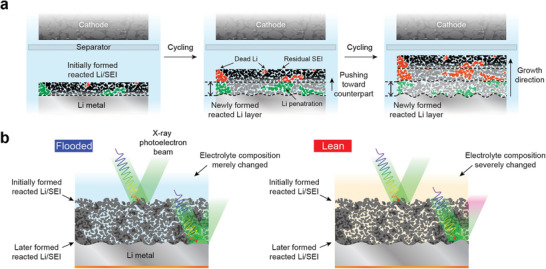
a) Scheme for pushing‐up growth mechanism of the reacted Li layer at the LMA. Initially developed reacted Li/SEI layer remains at the top whereas the newly formed SEI and reacted Li are formed beneath the preformed layer. b) Proposed XPS analysis to reveal the SEI chemistry after cycling. Particularly in a lean electrolyte, surface analysis of the native LMA surface can inform compositional changes of the electrolyte and the chemical evolution of the reacted Li layer over cycling.

To track the growth behavior of the reacted Li accumulated on the Cu foil, we obtained the cross‐sectional SEM images after selected cycles. Indeed, the growth rate of the reacted Li layer was observed in two distinct regions, which manifested as a change in the thickness and density of the reacted Li layer (Figure S2, Supporting Information).^[^ ^12^ ^]^ In the initial region, the LMA suffered from several side reactions such as electrolyte decomposition and “dead” Li formation with a high surface area, leading to the fast growth of the low‐density reacted Li layer. After a certain cycle, the apparent density of the reacted Li layer increases owing to the pressure in the cell, which induces an overpotential. We further investigated the detailed structural evolution related to the cell degradation by adopting the following approach (Figure S3, Supporting Information). After five cycles, a porous polyethylene terephthalate (PET) matrix was introduced on top of the partially grown reacted Li layer (Figure S3a). Subsequently, cycling was further carried out up to the 70th cycle to further grow the porous SEI (Figure S3b, Supporting Information). Given that the PET matrix was still observed regardless of the cycle number (Figure S3c,d, Supporting Information), a bottom‐up growth mechanism of the newly formed reacted Li layer is suggested as illustrated in **Figure** **1**a. The newly reacted Li/SEI forms directly on the native LMA surface while pushing up the previously formed reacted Li layer during repeated cycles. As a result, the initially formed reacted Li layer remains at the top region. Furthermore, the accumulated reacted Li layer engenders the cycling history, which can provide information about compositional changes within the electrolyte at each cycle. Considering that a side reaction‐triggered electrolyte contamination should be severe at lean electrolyte, the later formed SEI beneath the reacted Li layer could reflect the chemical evolution of Li/electrolyte interfaces and electrolyte composition after prolonged cycling (Figure 1b). Therefore, it is worth performing X‐ray photoelectron spectroscopy (XPS) measurements at the native LMA surface by peeling off the reacted Li layer, instead of the top surface, for accurate analysis of SEI chemistry after cycling.

### 2.2 Evolution of the Reacted Li Layer in Lean Electrolytes

**Figure 2:**
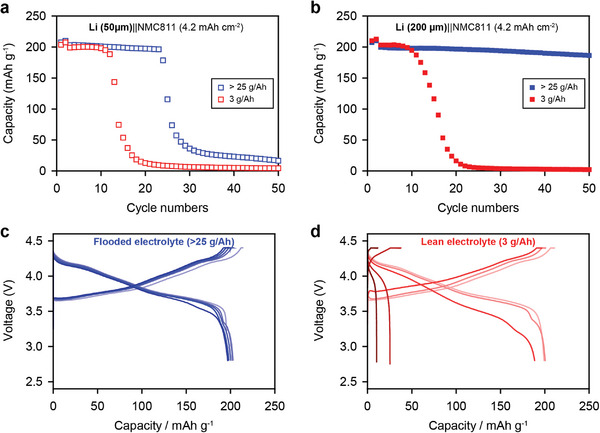
Cycling performance of Li||NMC811 cells with carbonate‐based electrolyte using ≈4.2 mAh cm^−2^ cathode coupling with a) 50 µm‐thick and b) 200 µm‐thick LMA under lean (3 g Ah^−1^) and flooded (>25 g Ah^−1^) electrolyte conditions, respectively. Evolution of voltage profiles of Li||NMC811 cells operated using a c) flooded and d) lean electrolyte.

Based on these findings, electrochemical cycling of LMBs was conducted to investigate the correlation between the cell failures and reacted SEI evolution with the electrolyte amount under practical conditions. In general, it is well‐known that a low E/C ratio accelerates premature cell failure due to a rapid accumulation of the reacted Li layer and rapid electrolyte depletion.^[^ ^10^, ^42^ ^]^ To understand these failure behaviors, it is essential to investigate the properties of the Li/electrolyte interface and their changes during cycling. **Figure** **2** shows the electrochemical performance of Li||NMC811 cells, including a high‐loading cathode (≈4.2 mAh cm^−2^) and thin and thick LMAs with conventional carbonate‐based electrolytes under lean and flooded electrolyte conditions, respectively. As shown in Figure 2a, the LMBs with the flooded electrolyte maintained their capacity for up to 25 cycles, whereas the LMBs with the lean electrolyte failed after 12 cycles. When the anode was replaced with a 200 µm‐thick LMA (Figure 2b), the cell achieved prolonged cycling in the flooded electrolyte owing to a Li excess of four times. By contrast, the cycling under lean electrolyte condition was still limited up to ≈12 cycles. While the Li||NMC811 cells with flooded electrolyte exhibited a negligible increase in the cell overpotentials (Figure 2c), the cell with the lean electrolyte exhibited a severe capacity drop and high overpotential immediately after 10 cycles (Figure 2d). An electrochemical impedance spectroscopy (EIS) study revealed that the cell containing the lean electrolyte exhibited an increased bulk resistance (*R*_b_), indicating that the electrolyte was severely depleted after 10 cycles (Figure S4, Supporting Information). Notably, Ni‐rich (>80%) NMC cathodes are often vulnerable to Ni^3+^ dissolution during cycling. Ni^3+^ ions released from the cathode could be migrated and get co‐deposited at the LMA surface, catalyzing the electrolyte decomposition.^[^ ^48^, ^49^, ^50^ ^]^ Such drawbacks can be mitigated by replacing the cathode with moderate Ni‐containing NMCs, such as NMC622. Nevertheless, the Li||NMC622 cells could not exceed 12 cycles (Figure S5, Supporting Information). When the electrolyte was refueled into the Li||NMC622 cell after cell failure, the original capacity was fully recovered. Thus, we concluded that a lack of electrolytes caused the earlier cycling failure. Consistently, the *R*_b_ shift of the lean electrolyte implies that electrolyte depletion is the main reason for cell failure. After reinjecting the electrolyte, *R*_b_ decreased again from 12.5 to 5.0 Ω (Figure S4b, Supporting Information). Regardless of electrolyte refueling, the interfacial resistance (*R*_inter_) between the lean and flooded electrolytes remained noticeable. These results indicate that additional interfacial issues occurred, except for the electrolyte depletion issues during cycling.

**Figure 3:**
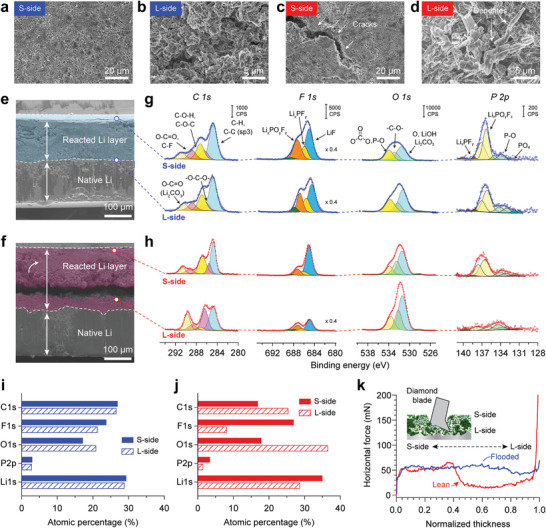
Postmortem analysis of the reacted Li layer. Top‐view SEM images of a,c) reacted SEI and b,d) native LMA surfaces for flooded (>20 g Ah^−1^) and lean (≈3 g Ah^−1^) electrolytes, respectively. e,f) Cross‐sectional SEM images of LMAs after cycling with flooded and lean electrolytes, respectively. High‐resolution XPS spectra obtained at separator (S) and LMA (L) sides of reacted SEI cycled at g) flooded and h) lean electrolyte. Atomic percentages of different elements on SEP and LMA sides of the reacted SEI obtained from the cells with i) flooded electrolyte, j) lean electrolyte. k) Peeling strength of reacted SEI of each LMAs. All samples were collected from Li (200 µm thickness)||NMC622 cells after the 10th cycle.

To reveal the effects of the electrolyte amount on the Li/electrolyte interface stability, the morphology of the LMA, including the reacted Li layer and native LMA, was investigated using SEM and XPS (**Figure** **3**). Considering that cross‐contamination triggered by the high‐Ni NMC cathodes could adversely affect interfacial reactions at the LMA side and lead to misinterpretation, SEM and XPS analyses hereafter were performed with the LMAs obtained from the Li||NMC622 cells. According to the aforementioned growth mechanism, the newly deposited Li and byproduct pushes up the existing layer and accumulates underneath the reacted Li layer. Thus, we examined the morphological and compositional changes at the top surface of the reacted Li layer (separator side, S‐side) and underneath the LMA surface (L‐side) after cycling. Under flooded electrolyte conditions, both the S‐ and L‐side surface morphologies exhibited compact and less dendritic Li growth because of the better uniformity and wettability of the electrolyte within the reacted Li layer (Figure 3a,b). In strong contrast, a non‐uniform interface was observed under lean electrolyte conditions (Figure 3c,d). The cycled LMAs showed large cracks at the S‐side surface of the reacted Li layer (Figure 3c) and intensive formation of “dead” Li on the native LMA (L‐side) surface under lean electrolyte conditions (Figure 3d). This difference implies that the structural evolution of the reacted Li layer was dominated by the electrolyte weight within the cells. Particularly in the lean electrolyte, the majority of the electrolyte can be trapped within the reacted Li layer owing to its porous nature, preventing it from reaching the original LMA, thereby leading to uneven Li plating and stripping. Furthermore, the LMAs with the flooded electrolyte attached well to the reacted Li layer on the native Li surface (Figure 3e), whereas the reacted Li layer developed in the lean electrolyte eventually delaminated from the surface (Figure 3f). Given that the chemical nature of the SEI is mostly governed by the electrolyte composition at each cycle, this finding implies that the SEI formed, between the reacted Li layer and original LMA after a certain number of cycles has chemically evolved. This evolution is very likely attributed to salt or solvent depletion at a lower E/C ratio.

The chemical evolution of the reacted Li layer was further verified by performing XPS analysis at both the S‐ and L‐sides. As shown in Figure 3e–g, the XPS spectra and atomic composition of the cell with the flooded electrolyte are similar on both the L‐ and S‐sides of the reacted Li layer. This implies that the chemical nature of the SEI at the flooded electrolyte was consistent regardless of the analysis of spot owing to a negligible change in the electrolyte composition. In stark contrast, significant changes in the composition of the SEI on both sides were observed in the case of the lean electrolyte (Figure 3h). The peak intensities of the F 1s and P 2p spectra, attributed to LiPF_6_ decomposition, were higher on the S‐side, implying that salt decomposition could have mostly occurred during the initial cycles. On the L‐side, the inorganic species (F 1s and P 2p spectra) were less abundant, whereas the atomic portion of the organic species (C 1s and O 1s spectra) increased. Such a compositional change indicates that LMAs under the lean electrolyte suffer from early Li salt depletion, which induces solvent‐derived SEI formation, such as polyether (C—O), carboxylate (O—C=O), and Li carbonate (Li_2_CO_3_) during later cycles. Further investigation of atomic compositions revealed a significant compositional change depending on the electrolyte amount. While the reacted Li layer, developed in the flooded electrolyte showed a negligible change regardless of the analyzing spot (Figure 3i), a dramatic compositional change in C, O, F, and P was confirmed in the case of the lean electrolyte (Figure 3j). At the L‐side, the proportion of inorganic components such as F 1s, P 2p was approximately twice that of the S‐side (i.e., for F 1s, S‐side: 26.9%, L‐side: 8.1% whereas for the P 2p, S‐Side 3.4%, L‐side 1.5%). On the L‐side, opposite trends in compositional changes were observed, indicating that organic components become dominant with an increase in the C 1s (16.9 to 25.3%) and O 1s (17.8 to 36.5%). XPS analysis of the upper and bottom spots of the reacted Li layer revealed compositional changes in the SEI formed at each cycle under lean electrolyte conditions. This could be a major reason for the structural deterioration of the reacted Li layer. Furthermore, a comparison of the atomic ratio and spectral distribution accounts for the electrolyte decomposition events during the cycling period. While the SEI formed at the initial stage was mostly derived from the salt; the SEI at the later stage was mostly governed by organic solvents, which is likely due to fast depletion of the Li salt. Furthermore, such electrolyte composition could induce the deterioration of cathode‐electrolyte interphase (CEI). As confirmed in the XPS study at the cycled cathodes harvested from different cells, dramatic peak evolution was confirmed in all spectra, and the transition metal (e.g., Ni, Mn, Co) was not detected in the case of lean electrolyte (Figures S6 and S7, Supporting Information), which indicates the thickening of CEI in the lean electrolyte owing to severe contamination. This supports that the electrolyte composition changes triggered by LMA cycling was significant at the lean electrolyte and the cathode surface can be deteriorated.

The mechanical integrity of the reacted Li layer was quantitatively examined using a surface and interfacial cutting analysis system (SAICAS) placed in a glove bag filled with N_2_ (Figure S8, Supporting Information). SAICAS is a versatile tool to measure the mechanical properties, such as the cohesive and adhesive strength of any composite layer at a specific spot by controlling the microblade depth.^[^ ^51^, ^52^ ^]^ Figure S8b,c (Supporting Information) shows a schematic illustration of the measurement process corresponding to the optical image during the SAICAS measurement. The mechanical properties were confirmed by an in‐depth peeling of the reacted Li layer beneath the Cu surface. As shown in the SAICAS measurement profiles (Figure 3k), there are noticeable differences in the horizontal force between the top and bottom interfaces under the lean electrolyte condition. While the reacted Li layer under the flooded electrolyte exhibited a constant peeling force (≈50 mN) in the vertical direction, a relatively weak cutting force (≈20 mN) was measured near the interface in a lean electrolyte. Therefore, it was confirmed that there were relatively more organic species with a low mechanical strength in the vicinity of the interface, which led to the delamination of the reacted Li layer.

### 2.3 Evolution of the Reacted Li Layer with LHCE

**Figure 4:**
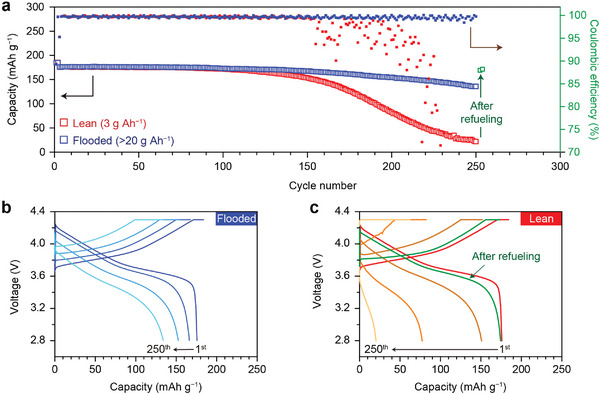
Comparison of the electrochemical performance of Li (50 µm)||NMC622 (4.0 mAh cm^−2^) cells with LHCE between lean and flooded electrolyte conditions. a) Cycling performance of Li||NMC622 cells operated at C/3 charge and discharge (1 C = 4.0 mA cm^−2^). Voltage curves of Li||NMC622 cells at the selected cycles (1st, 150th, 200th, 250th) under b) flooded and c) lean electrolyte conditions.

The carbonate‐based electrolyte induced dramatic growth and evolution of the reacted Li layer owing to its poor compatibility with LMA. Although dynamic evolutions were clearly observed, further verification using more compatible electrolytes with LMA is necessary for practical applications. Indeed, the LHCE concept proposed by the Pacific Northwest National Laboratory group has been suggested as a basic electrolyte platform for stabilizing LMAs.^[^ ^39^, ^53^, ^54^ ^]^ The stable cycling of LMBs with LHCEs has been validated in a pouch cell configuration under stringent conditions.^[^ ^41^ ^]^ Thus, the LHCE (LiFSI/DME/TTE, 1:1.2:3, by mol) was preferentially exploited to investigate the chemical evolution of the reacted Li layer in the lean electrolyte (**Figure** **4**). A much longer cycle, compared to the carbonate‐based electrolytes, was achievable owing to the highly stable characterization of LHCE (Figure 4a). Despite the superiority of LHCEs toward LMAs, the LMB in the lean electrolyte, the LHCEs exhibit a limited cycle performance compared to the flooded electrolyte condition (Figure 4b,c). The electrolyte reinjection test proved that the amount of electrolyte was still the main cause of cell failure (Figure 4c). Therefore, these results imply that an inhomogeneous reaction can occur because of the change in the electrolyte composition under the lean electrolyte conditions even in the LHCE system.

To confirm the dependence of the structural evolution of the reacted SEI on the amount of electrolyte, the LMAs after cycling were investigated using SEM (Figure S9, Supporting Information). After the 40th cycle, similar morphologies of the top surface were observed for the lean and flooded electrolyte conditions (Figure S9a,d, Supporting Information). Owing to the merits of LHCE, it forms a stable SEI toward Li metal, which leads to fewer side reactions and electrolyte consumption even at low E/C ratios (≈3 g Ah^−1^). However, some differences were observed in the remaining native Li (Figure S9b,e, Supporting Information). The native Li cycled in the flooded electrolyte showed an evenly Li stripped morphology (Figure S9e, Supporting Information), whereas unused Li was observed under lean electrolyte conditions (Figure S9b, Supporting Information). The cross‐sectional SEM image clearly showed the irregular Li plating/stripping properties of the LMAs under lean electrolyte conditions (Figure S9c,f, Supporting Information).

**Figure 5:**
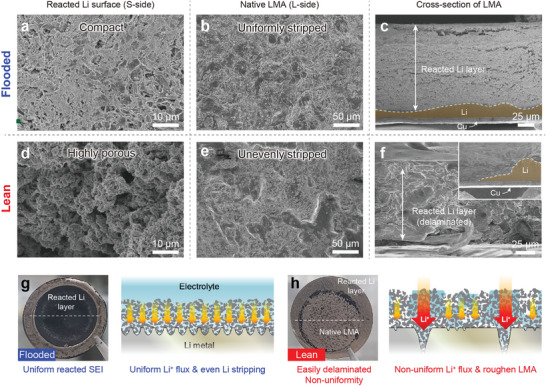
Structural evolution of the reacted Li layers developed at the LMAs in Li||NMC622 cells with LHCE after 120 cycles. Top‐views of the reacted Li layer and native LMA under the flooded a,b) and lean d,e) electrolyte conditions. Cross‐sectional SEM images of cycled LMA under c) flooded and f) lean electrolyte conditions (Inset: LMA laminated Cu side). A schematic and digital camera image of the reacted Li layer in the g) flooded and h) lean electrolytes.

Obvious differences were confirmed after 120 cycles when the capacity degradation started. The reacted Li morphologies in the flooded electrolyte exhibited more compact Li dendrites and uniform Li dissolution (**Figure** **5**a,b). At low E/C ratios, even after 120 cycles, Li dissolution at local spots was consistently confirmed on the surface of the native LMA, which is expected to induce highly porous Li dendrite growth (Figure 5d,e). In the cross‐sectional view, the LMAs in the flooded electrolyte exhibited a lower thickness of the reacted Li layer with densely packed morphologies and maintained their native Li layer even after prolonged cycling (Figure 5c). On the other hand, the lean electrolyte case has an unevenly porous Li dendrite morphology similar to that of a sponge (Figure 5f). The evolution of the reacted Li layer with respect to the electrolyte amount can be explained by the electrolyte wetting and distribution at the LMAs surface. The flooded electrolyte can fully cover the LMA surfaces, leading to uniform Li plating/stripping and compact morphologies of the reacted Li layer (Figure 5g). In contrast, a relatively small number of electrolytes were partially trapped as the reacted Li layer became thicker, resulting in a non‐uniform distribution and local electrolyte depletion at the Li/electrolyte interface (Figure 5h). Moreover, the inactive TTE diluent, which could not transport Li ions, occupied the high volume portion of the electrolyte, further exacerbating such electrolyte distribution issues. The uneven interface between the LMA and the reacted Li layer, especially at lower E/C ratios, exacerbates the uniformity of the Li^+^ flux at the LMA, resulting in severe Li dendrite growth through the reacted Li layer where the electrolyte is filled. This result suggested that a remaining significant non‐uniformity of the electrolyte distribution at the interface at lower E/C ratios, which may infer continuous electrolyte loss upon cycling, leading to poor electrolyte utilization and depletion.

**Figure 6:**
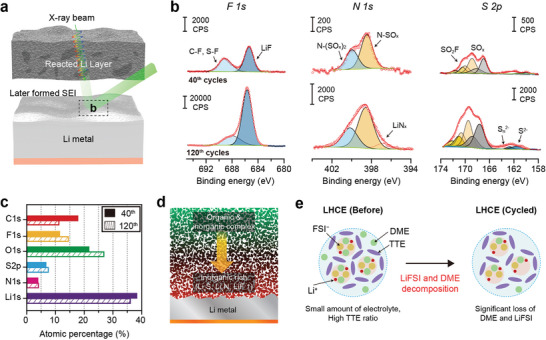
Chemical evolutions of the reacted Li layers facing the LMAs after 40, 120 cycles. a) Local spot of XPS measurement to examine the later formed SEI. b) High‐resolution XPS spectra of F 1s, N 1s and S 2p spectra after 40, 120 cycles. c) Quantification of atomic concentration after cycling. Schematic of d) chemical evolutional change of reacted Li layer and e) change in electrolyte composition during cycling.

To determine the effect of the electrolyte compositional change on the chemical evolution upon cycling, we analyzed the reacted Li layer in cycled Li||NMC622 cells with LHCE using XPS. As suggested earlier, the region beneath the reacted Li can provide information on the electrolyte compositional change and SEI properties at the selected cycle (**Figure** **6**a). For this, we unraveled the embedded region beneath the reactive Li layer by peeling the porous SEI at the 40th and 120th cycles for investigating its chemical properties (Figure 6b). As a result, the intensities of LiF, Li_3_N, and sulfur compounds, derived from FSI‐ decomposition, were increased during cycling. Moreover, similar N and S atomic ratios were observed regardless of the cycle number, suggesting that the LiFSI salt continuously contributed to the SEI formation reaction during the prolonged cycling (Figure 6c). In contrast, the intensity of the C 1s peak at the 120th cycle is lower than that at the 40th cycle. These findings suggest that the main solvent (e.g., DME) depletion occurred earlier, and thus, the solvent contribution to the reacted Li layer development decreased during the later cycles. Hence, compositional evolutions in the reacted Li layer and the electrolyte still occurred even with a highly compatible LHCE, which leads to cell failure, as illustrated in Figure 6d, for LMAs at low E/C ratios of LHCE. During prolonged cycling, the continuous salt (e.g., FSI anion) and DME decomposition triggered the collapse of the LiFSI/DME complex composed of poor organic compounds of the SEI at a later cycle.

Consequently, two main issues correlate the structural and chemical evolution of the reacted Li layer with the electrolyte compositional change at lower E/C ratios. First, a higher ratio of inactive TTE within lean electrolyte could rather impede the uniform distribution of the LiFSI/DME complex toward the LMA surface, which leads to a structurally non‐uniform Li/electrolyte interface. Second, the consumption of the LiFSI salt and DME solvent occurred continuously during cycling. In particular, the depletion of the DME solvent became more severe in later cycles. Consequently, the unique solvation structure of LHCE collapsed, reducing the LiFSI and DME portions and increasing the proportion of inactive TTE diluent during cycling (Figure 6e). This indicates that changes in the electrolyte composition trigger the formation of a structurally non‐uniform Li/electrolyte interface. It is worth noting that a design strategy for improving the compatibility of LHCE is required to overcome the structural and chemical problems at a low E/C ratio. In this regard, we proposed new composition design strategies by increasing the LiFSI/DME complex within a specific volume and reducing the inactive TTE ratio to overcome various issues at lower E/C ratios.

**Figure 7:**
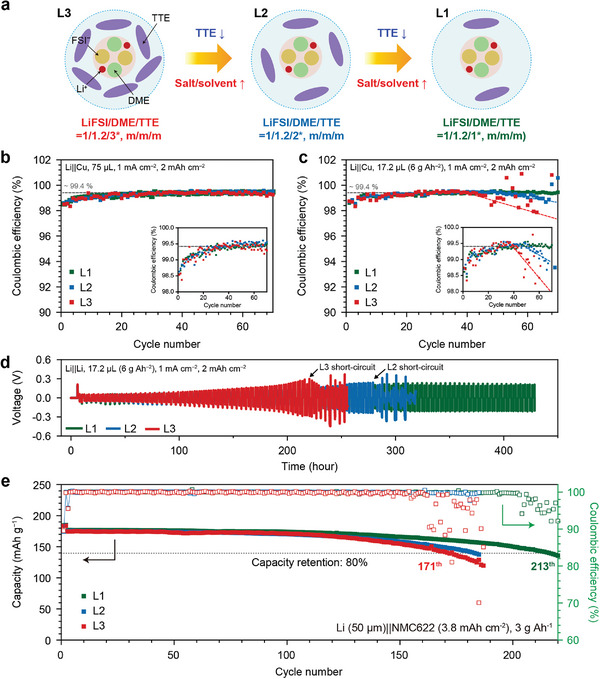
Electrochemical characteristics of LHCEs with different TTE ratios. a) Schemes of solvation structure of redesigned LHCEs (L3, L2, and L1). Li||Cu cell cycling with different LHCEs at controlled electrolyte amounts; b) flooded (75 µL) and c) lean (17.2 µL) electrolytes. Insets show a magnified view of Li CE variations. d) Comparison of Li||Li cell cycling stability for three LHCE under the lean electrolyte condition. e) Cycling performance of Li||NMC622 cells with different electrolytes under practical conditions. All cells were composed of thick cathode (3.8 mAh cm^−2^), thinner LMA (50 µm), and lean electrolyte (3 g Ah^−1^). All Li||NMC622 cells were charged at C/3 and discharged at C/3 after two formation cycles at C/10 charge and discharge.

To reveal the effects of reducing the diluent ratio, three different LiFSI‐1.2DME‐xTTE (where *x* = 1, 2, and 3, molar ratios are; denoted as L1, L2, and L3, respectively) electrolytes were designed as shown in **Figure** **7**a. When the molar ratio of inactive TTE was reduced from 3 to 1, the volume percentage of LiFSI and DME increases from 31% to 58%. The HFE diluents have been introduced to overcome several challenges related to HCEs, such as high viscosity and insufficient wettability of the separator and thick electrodes.^[^ ^34^, ^35^ ^]^ Although reducing the TTE ratio could be less beneficial for reducing the viscosity and wettability enhancement, the physical properties of the electrolytes were still acceptable even at moderate dilution with a lower amount of TTE (Figure S10 a,b, Supporting Information). To validate the compatibility of the designed electrolyte with LMA and cathode, Li||Cu cell cycling and linear sweep voltammetry (LSV) tests were conducted. Regardless of the TTE ratio in the electrolyte, all electrolyte compositions exhibited similar CEs around 99.4% in Li||Cu cell tests with the flooded LHCE (E/C ratio, 20 g Ah^−1^) (Figure 7b) and comparable oxidation stability in LSV tests (Figure S11, Supporting Information). The oxidative and reductive stabilities of electrolytes composed of the same components are predominantly determined by their solvation structures. Because the TTE has negligible Li solvation ability,^[^ ^42^ ^]^ the degree of TTE dilution did not affect its compatibility with LMA and oxidative stability.

Despite compromising the electrolyte viscosity, reducing the degree of TTE dilution is to occupy more “active” components (LiFSI and DME) within the LHCE and further delay their depletion over prolonged cycling. Thus, the depletion rate could be lowered by decreasing the TTE molar ratio, influencing the cycling stability. Given that all electrolytes share a similar Li^+^ solvation environment, all Li||Cu cells behaved consistently in the earlier stage of cycling, showing similar voltage curves (Figure S12, Supporting Information). Besides, the initial Li CEs and stabilized CE were consistent at ≈98.7% and 99.4%, respectively. Nonetheless, the most striking difference was the cycling persistence of the Li||Cu cells at a controlled E/C ratio (≈6 g Ah^−1^) (Figure 7c). The cell with the L3 electrolyte showed the earliest signs of CE fading, followed by the L2 electrolyte, whereas the L1 electrolyte exhibited excellent CE retention over 100 cycles, even under the lean electrolyte condition. Similarly, it was confirmed that the Li||Li cell with the L1 electrolyte could secure cycling stability as the dilution degree decreases under the lean electrolyte condition (Figure 7d). In postmortem SEM images (after the 70th cycle), the reacted Li layer formed at the L1 electrolyte exhibited reduced cracks by mitigating the electrolyte shortage. Moreover, the reacted Li layer thickness was reduced at the L1 electrolyte compared to others (Figure S13, Supporting Information), suggesting that reducing the TTE dilution degree is beneficial to alleviate anode swelling and delay in the electrolyte loss‐driven cell failure. The XPS analysis further revealed that the L3 electrolyte exhibited a rapid decrease in the C atomic ratio, despite the relatively higher atomic ratio of F, N, S, and Li (Figure S14, Supporting Information). This indicates that SEI building becomes abnormal as the LiFSI and DME are depleted earlier in the L3 electrolyte. By contrast, the reacted Li layer developed at the L1 electrolyte retained a higher C atomic ratio but relatively less FSI‐derived atoms, suggesting that the SEI evolution could be mitigated by enriching the active components. To fully verify the effect of increasing the proportion of active LiFSI/DME complexes, Li||NMC622 cells with different electrolytes were tested under practical conditions. As shown in Figure 7e, a lower TTE ratio resulted in a better cycle stability, even at lower E/C ratios (3 g Ah^−1^). While the cells with L3 electrolyte showed capacity fading relatively early with a capacity retention of 80% at the 171st cycle, the L1 electrolyte exhibited an enhancement in the cycling performance (80% retention at the 213th cycle). These results demonstrate that reducing the diluent can help enhance the cycling stability of LMBs owing to the abundance of salt and main solvent in a specific volume of the electrolyte.

## 3 Conclusion

In this study, the “pushing up” growth of the reacted Li layer and its structural and chemical evolutions upon cycling were elucidated, thereby revealing the anode swelling behavior in the LMBs. In contrast to no obvious changes in the chemical composition of the top surface, the chemical evolutionary behavior underneath the reacted Li layer could be an important indicator of electrolyte compositional changes. In a lean electrolyte (E/C ratio, ≈3 g Ah^−1^), poor electrolyte distribution causes uneven growth of the reacted Li layer, resulting in mechanical failures, such as cracks and delamination. Furthermore, it was clearly demonstrated that the chemical evolution in the lean electrolyte was more dramatic owing to a drastic compositional change. For the corrosive carbonate electrolyte, the organic components were dominantly formed beneath the porous SEI layer due to the early depletion of Li salt anions, leading to a poor mechanical stability near the LMA surface as confirmed by the SAICAS analysis. By further exploiting a highly compatible electrolyte, e.g., LHCE, a similar phenomenon was observed under the lean electrolyte condition. Notably, it was revealed that the FSI anions and DME solvents coordinated with Li^+^ within the LHCE and were rapidly depleted during the initial cycles in contrast to the flooded LHCE (E/C ratio, 20 g Ah^−1^). Considering the predominant decomposition of the major species (LiFSI/DME) at a lower E/C ratio, increasing their proportion while maintaining the overall solvation structure effectively retarded the electrolyte depletion. As a result, a new LHCE composition tailored by reducing the ratio of inert diluent (from L3 to L1) demonstrated prolonged cycling (171→213 cycles at 80% retention) of Li||NMC622 cell without compromising the Li CE (≈99%) and high voltage stability (≈4.4 V). Based on the finding that lean electrolytes can suffer from severe compositional changes upon cycling, understanding the chemical/structural evolutions of the reacted Li layers underneath can provide new insights into a new electrolyte design for longer cycling of practical LMBs.

## 4 Experimental Section

### Preparation of Electrodes and Electrolytes

The slurries of the LiNi_0.6_Mn_0.2_Co_0.2_O_2_ (NMC622) and LiNi_0.8_Mn_0.1_Co_0.1_O_2_ (NMC811) cathodes were prepared by mixing 96 wt.% NMC622 or NMC811, 2 wt.% poly(vinylidene fluoride) (PVDF) and 2 wt.% Super‐P in *N*‐methyl‐2‐pyrrolidone (NMP) solvent. Then, the slurry was cast on an Al foil using a doctor blade and subsequently dried at 160 °C (1.5 h) and 60 °C (overnight) in vacuum. The areal capacity of the NMC622 and NMC811 cathodes was controlled at ≈3.8–4.2 mAh cm^−2^. The electrode density was adjusted to 2.8 g cm^−3^ via calendaring. For the LSV study, the Super‐P/PVDF electrode was prepared by casting the Super‐P/PVDF slurry with NMP onto an Al foil and then drying at 130 °C (2 h). The mass loading of Super‐P was ≈0.6 mg cm^−2^. Li foil (200 µm thickness) was purchased from China Energy Lithium, China, and a 50 µm Li‐metal foil was purchased from Honjo Metal, Japan. For carbonate‐based electrolytes, 1.0 m lithium hexafluorophosphate (LiPF_6_) in ethylene carbonate (EC)/diethyl carbonate (DEC) (1:1 by volume) was used in the tests to investigate the reacted Li layer growth mechanism, 1.15 M LiPF_6_ and a mixture of EC and ethyl methyl carbonate (EMC) (3:7 by volume) with 2 wt.% vinylene carbonate (VC) additive was for Li||NMC622 cycling. All chemicals were battery‐grade and were purchased from Enchem, South Korea. The LHCE was prepared by dissolving Lithium bis(fluorosulfonyl)imide (LiFSI, Enchem) in 1,2‐dimethoxyethane (DME, Sigma–Aldrich) and 1,1,2,2‐tetrafluoroethyl‐2,2,3,3‐tetrafluoropropyl ether (TTE, SynQuest Labs, USA) with a molar ratio of 1:1.2:3 in an Ar‐filled glovebox (<1 ppm H_2_O and O_2_). The TTE molar ratio was controlled at 1, 2, and 3 to redesign the composition. All solvents were dried using molecular sieves (Aldrich) before use.

### Electrochemical Measurements

Coin cells (CR2032) were assembled in a glove box. All cell tests were performed using a battery cycler (WBCS3000L, WonATech, South Korea). To investigate electrolyte consumption and reacted Li layer growth mechanism, Li||Cu cell cycling was performed with a Li plating capacity of 1 mAh cm^–2^ and then fully stripped to 2.0 V (vs Li/Li^+^) at a constant current (0.5 mA cm^–2^). Li||Cu cells were pre‐cycled (1 cycle) before the main cycling at a current density of 0.1 mA cm^−2^ and an areal capacity of 1 mAh cm^−2^. For Li||NMC622 cells, the LMA (area: 2.01 cm^–2^), polyethylene separator (area: 2.83 cm^–2^), and NMC622 (area: 1.13 cm^–2^) cathode were assembled. The cells were cycled two times at 0.1 °C (≈0.4 mA cm^−2^) over a voltage range of 2.8–4.3 V for the formation process at 25 °C. Subsequently, cycling tests were performed at C/3 charge and discharge current density (constant current/constant voltage (CC/CV) and CC modes for charge and discharge steps, respectively) within a voltage window of 2.8–4.3 V. To avoid the corrosion of the cathode cases by the ether‐based electrolytes, Al‐clad cathode cases were used, and an additional piece of Al foil (2.83 cm^−2^) was placed underneath the cathode when using LHCEs. Electrochemical impedance spectroscopy (EIS) was performed using a potentiostat (VMP‐300, BioLogic, France). The frequency range was 5 MHz to 50 mHz with an AC amplitude of 10 mV. For the Li CEs measurements of the LHCEs in Li||Cu cells, a fixed amount of Li metal (1 mAh cm^−2^) were plated on the Cu electrode and subsequently stripped up to a cut‐off voltage of 1.0 V at a current density of 1 mA cm^−2^. LSV tests were performed from OCV to 6 V versus Li/Li^+^ at 0.5 mV s^−1^. The electrolyte amounts were controlled at ≈3 g Ah^−1^ (lean, 13 µL) and 75 µL (flooded) for Li||NMC622 cell tests. Excess electrolyte was injected into each coin cell unless otherwise specified.

### Characterization

After electrochemical tests, the cells were disassembled, and each electrode sample was collected. All electrode samples were washed strictly with DMC or DME for carbonate‐based electrolytes or LHCE and dried in a vacuum chamber at 25 °C for 12 h. To observe the behavior of porous SEI growth, the coin cells were reassembled using cycled Cu electrode and PET fiber mat (W2BF0310, NKK, Japan) and then re‐cycled. The morphological features of the LMAs were investigated using FE‐SEM (SU8020, Hitachi, Japan) at acceleration voltages of 3 or 5 kV. To examine the chemical evolutions of SEI, the separator (S) and LMA (L) sides of reacted SEI were analyzed using XPS (ESCALAB 250Xi, Thermo Fisher Scientific, USA). For sample preparation, the reacted Li/SEI samples were rinsed by completely soaking in pure DMC and DME solvents when carbonate and LHCE were used for the cycling test, respectively. This process was repeated twice to remove the residual salt and electrolyte solvents. Subsequently, the samples were vacuum‐dried overnight. To analyze the L‐side surface of the reacted Li layer, it was detached from the LMA using polyimide tape. The sample‐mounted XPS holder was carried using the PP container with air‐tight sealing and then quickly transferred into the analyzing chamber. All sampling procedures, sample mounting, and container sealing were performed in an Ar‐filled glove box to prevent the contamination of samples from ambient air. The mechanical strength of the porous SEI was analyzed using surface and interfacial cutting analysis system (SAICAS, Daipla Wintes, Japan) in a glove bag filled with N_2_. A diamond blade (width: 1 mm, rake angle: 20°, clearance angle: 10°) was used to cut the porous SEI and measure the cutting force. While cutting the porous SEI, a blade moved horizontally at 2 µm s^−1^ while maintaining a vertical force of 0.5 N.^[^ ^55^ ^]^

## Conflict of Interest

The authors declare no conflict of interest.

## Supporting information

Supporting Information

## Data Availability

The data that support the findings of this study are available in the supplementary material of this article.
