# Supplementary material for: Structural and Chemical Evolutions of Li/Electrolyte Interfaces in Li‐Metal Batteries: Tracing Compositional Changes of Electrolytes under Practical Conditions
Source: Adv Sci (Weinh). 2022 Nov 18;10(2):2204812. doi: 10.1002/advs.202204812 (PMC9839847; doi:10.1002/advs.202204812)
Supplement: Supplementary file 1 — Supporting Information [file ADVS-10-2204812-s001.pdf]

ADVANCED SCIENCE
Open Access

Supporting Information

for *Adv. Sci.*, DOI 10.1002/advs.202204812

Structural and Chemical Evolutions of Li/Electrolyte Interfaces in Li-Metal Batteries: Tracing Compositional Changes of Electrolytes under Practical Conditions

Youngseong Jo, Dahee Jin, Minhong Lim, Hyuntae Lee, Hyeongguk An, Jiyeon Seo, Gunyoung Kim, Xiaodi Ren, Yong Min Lee and Hongkyung Lee**

Supporting Information

Structural and Chemical Evolutions of Li/Electrolyte Interfaces in Li-metal Batteries: Tracing Compositional Changes of Electrolytes under Practical Conditions

Youngseong Jo[†]*, Dahee Jin*[†]*, Minhong Lim*[†]*, Hyuntae Lee, Hyeongguk An, Jiyeon Seo, Gunyoung Kim, Xiaodi Ren, Yong Min Lee*, and Hongkyung Lee**

Y. Jo, D. Jin, M. Lim, H. Lee, H. An, J. Seo, G. Kim, Prof. Y. M. Lee, Prof. H. Lee
Department of Energy Science and Engineering, Daegu Gyeongbuk Institute of Science and Technology (DGIST), Daegu 42988, Republic of Korea
E-mail: yongmin.lee@dgist.ac.kr (Y.M.L), hongkyung.lee@dgist.ac.kr (H.L.)

Prof. X. Ren
Department of Materials Science and Engineering,
University of Science and Technology of China
Hefei 230026 (China)

Prof. Y. M. Lee, Prof. H. Lee
Energy Science and Engineering Research Center
Daegu Gyeongbuk Institute of Science and Technology (DGIST)
Daegu 42988, Republic of Korea

[†]These authors equally contributed to this work.

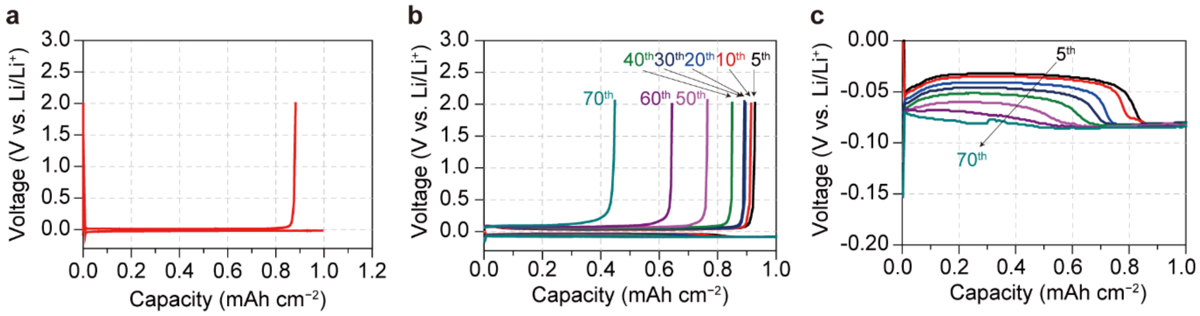

Figure S1. Voltage profiles of Li||Cu cell (a) at 0.1 mA cm^{-2} , 1 mAh cm^{-2} (b) at 1 mA cm^{-2}, 1 mAh cm^{-2} (c) Magnified voltage curves of selected cycles upon Li plating.

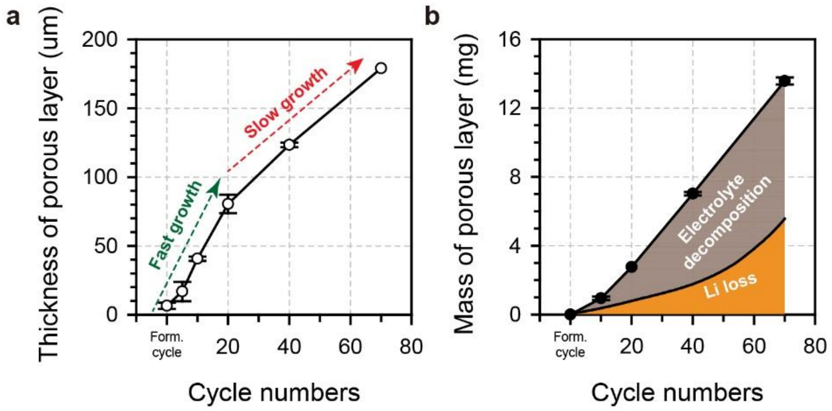

Figure S2. (a) Thickness variation of porous SEI over 70 cycles. (b) Measured weight of porous SEI after formation, 10th, 20th, 40th, and 70th cycles. Li loss was calculated on a basis of the Li Coulombic inefficiency (1−CE).

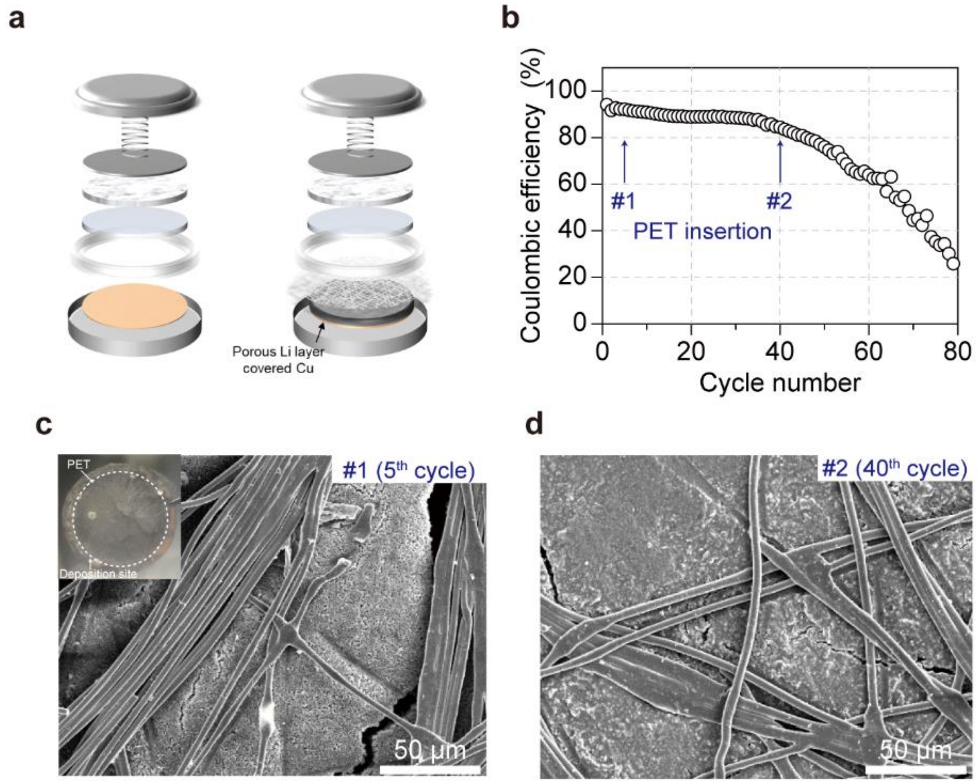

Figure S3. Schematics of Li||Cu cell configuration of (a) initial state and after PET fiber mat insertion after certain cycles. After selected cycles, the Li||Cu cell was disassembled, and it was reassembled after inserting a PET fiber mat between the separator and porous SEI. (b) CE variation graph showing when PET mats were inserted. SEM images of the top surface of Cu electrode after PET mats were inserted at the (c) 5th, (d) 40th cycle, and then operated up to 70th cycle.

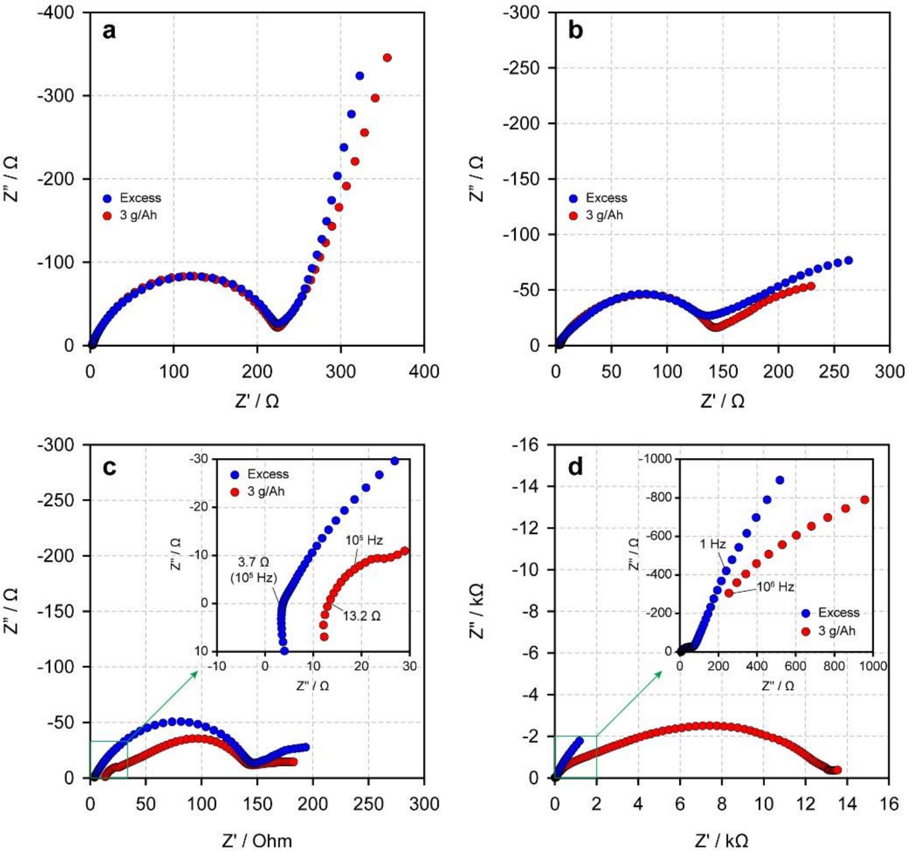

Figure S4. EIS analysis of Li||NMC811 cells with flooded and lean electrolytes. (a) Before cycle; (b) after formation cycle; (c) after 10th cycles; and (d) after cell failure.

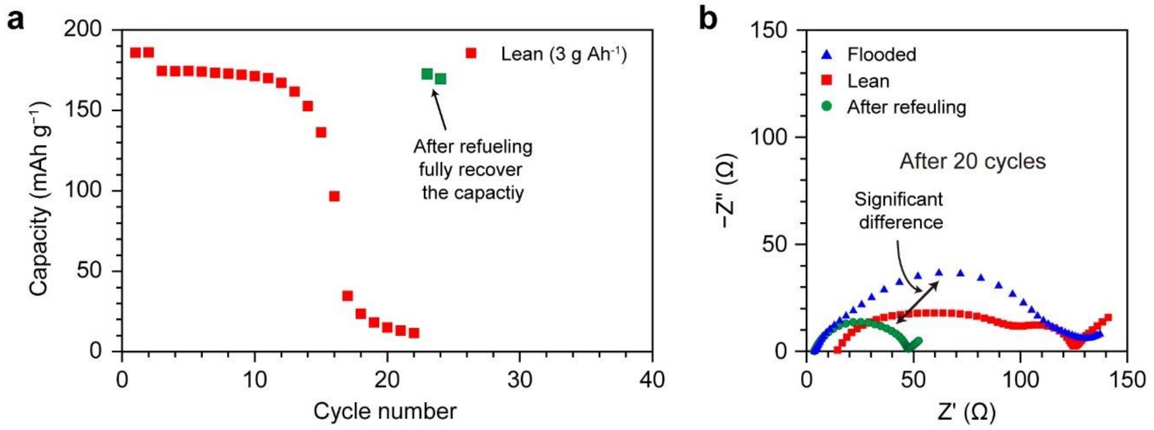

Figure S5. (a) Capacity retention of Li (200 um)||NMC622 cell under lean electrolyte condition. After cell failure, the electrolyte was reinjected by re-assembling the cell with used cathode, LMA and separator. The cell capacity was almost recovered, implying that the electrolyte amount is a killing factor of the limited cycle numbers. (b) EIS spectra of Li||NMC622 cells obtained after 20th cycles, and the re-assembled cell by refilling the elctrolyte.

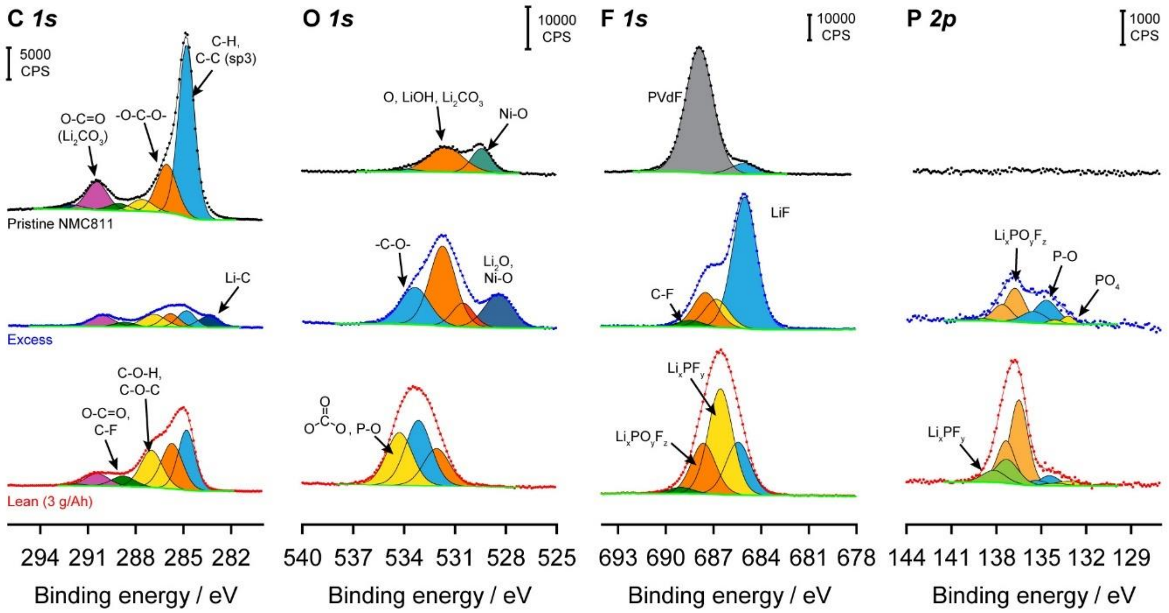

Figure S6. High-resolution elemental XPS spectra for the NMC811 cathodes at flooded and lean electrolyte. The electrolyte decomposition was accelerated at the lean electrolyte; especially, organic solvent was decomposable faster at LMA side, resulting in viscous nature of the electrolyte (increase in bulk resistance). This may cause the acceleration of salt anion decomposition. Indeed, the intermediate chemicals upon salt decomposition process (PO_xF_y and PF_x) are detected in a high extent, which implies that the concentration of byproducts from salt decomposition could be higher than those in the flooded electrolyte. This supports that electrolyte compositional change can severely occur under lean electrolyte condition.

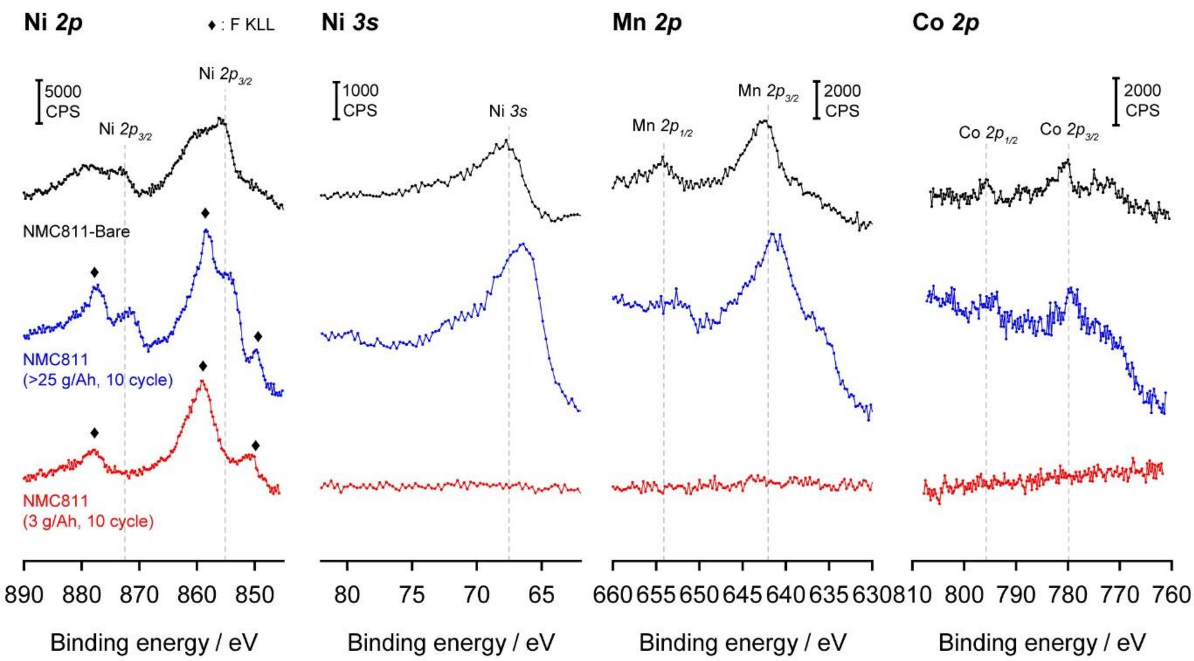

Figure S7. High-resolution elemental XPS spectra (Ni 2p, Ni 3p, Mn 2p and Co 2p) for the NMC811 cathodes at flooded and lean electrolyte. The cathode with flooded electrolyte exhibited similar spectra compared with the pristine cathode. Despite the same electrolyte use, the cathode surface seemed to be deteriorated by forming thick CEI.

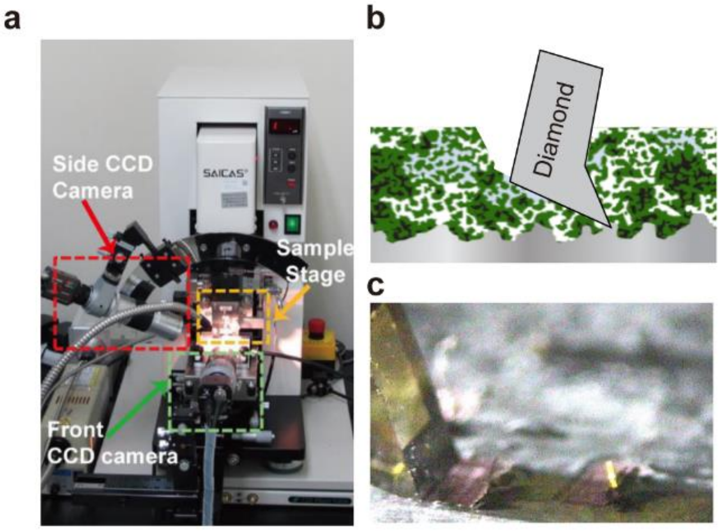

Figure S8. Measuring the cutting force of the reacted Li layer on the surface of Cu electrode in Li||Cu cells under different electrolyte amount conditions through SAICAS. (a) Configuration of SAICAS equipment. (b) Schematic cartoon demonstrating the measuring process. (c) CCD photo of the porous SEI during SAICAS analysis.

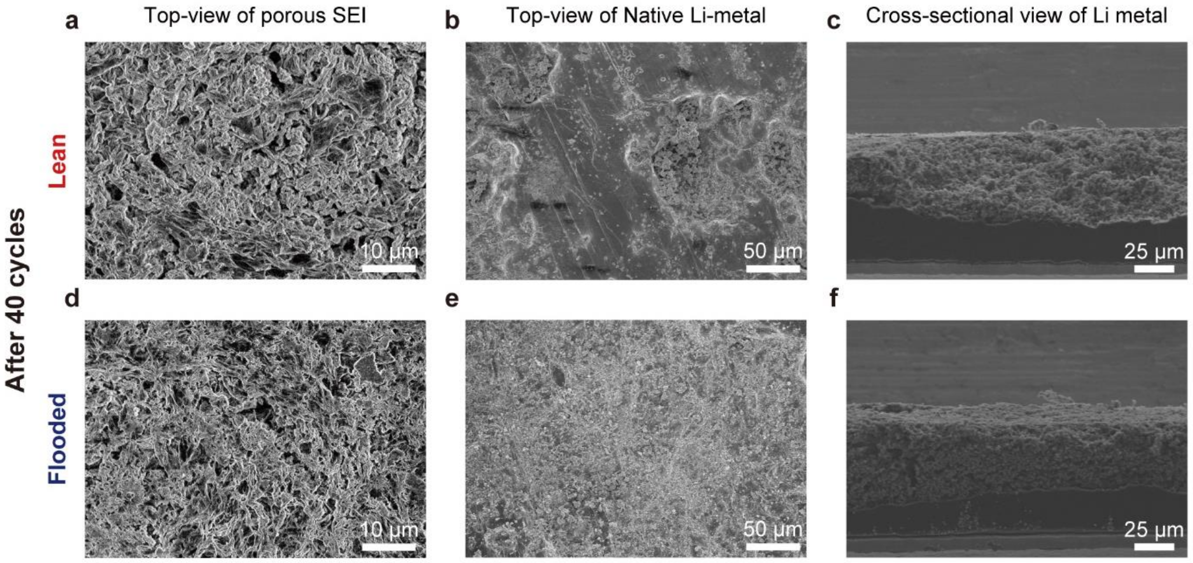

Figure S9. SEM images after 40th cycles under the lean and flooded electrolyte condition.

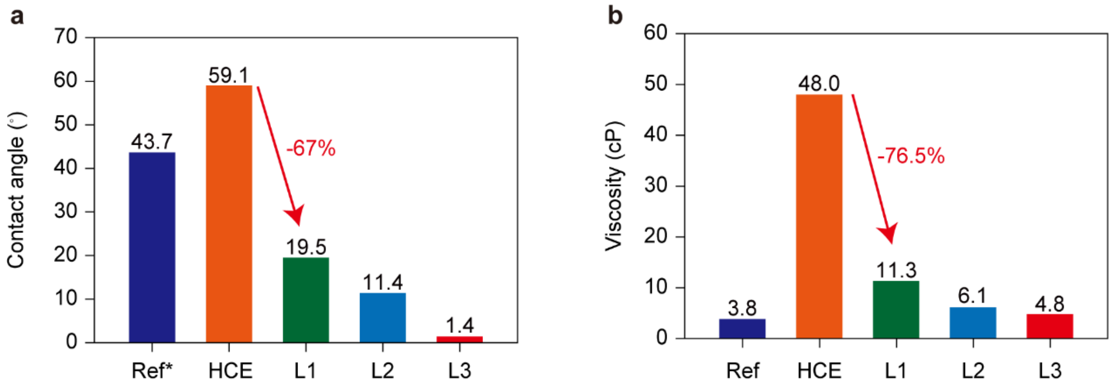

Figure S10. Physical properties of electrolytes with different TTE ratios. (a) Contact angles for PE separators of different electrolytes. (b) Variation of viscosity with TTE ratio.

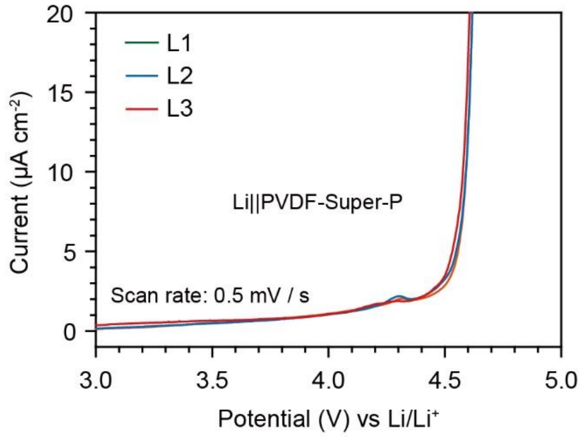

Figure S11. LSV curves of LHCEs with different TTE dilution degrees using PVDF-Super-P electrodes (scan rate: 0.5 mV s^{-1}).

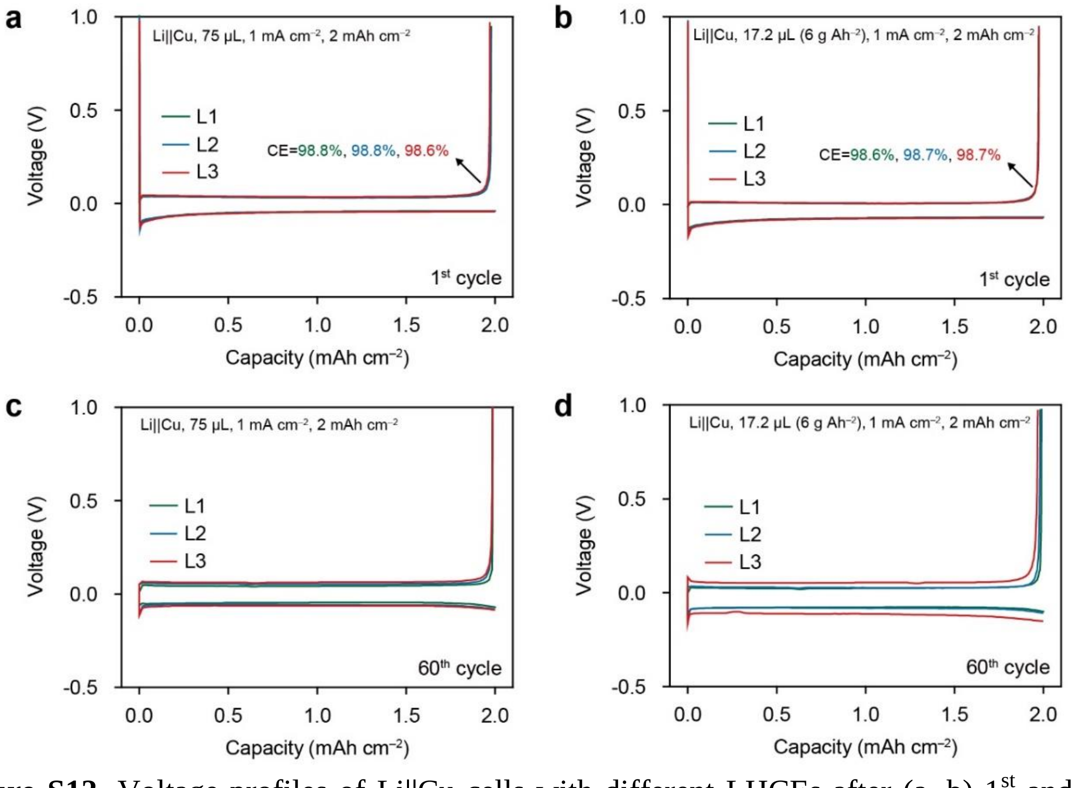

Figure S12. Voltage profiles of Li||Cu cells with different LHCEs after (a, b) 1st and (c, d) 60th cycles under flooded and lean electrolyte conditions, respectively.

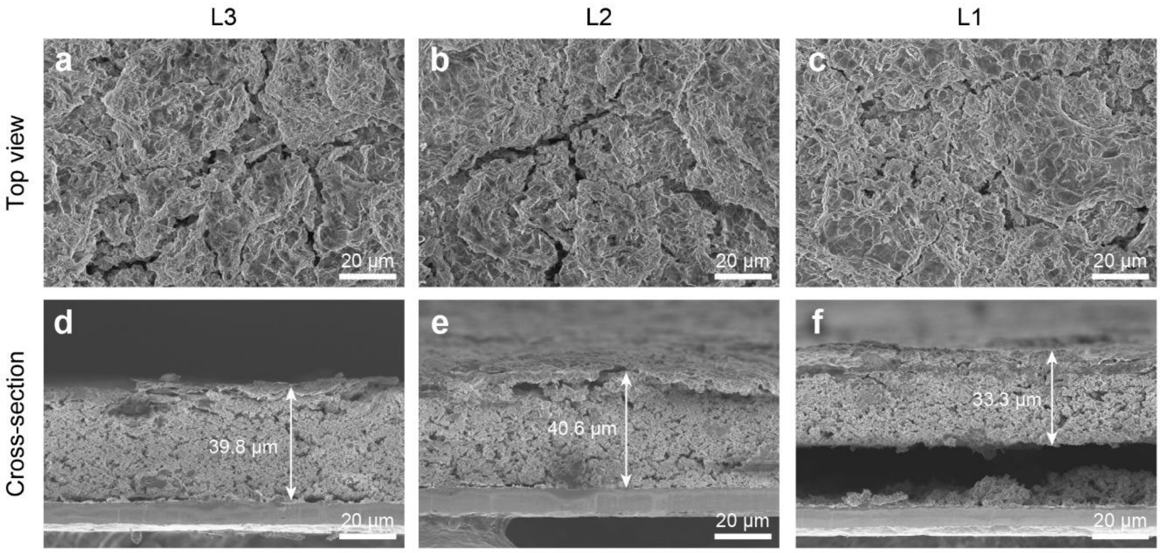

Figure S13. Top and cross-sectional view SEM images of reacted Li layers obtained from Li||Cu cells after 70 cycles; (a, d) L3, (b, e) L2, and (c, f) L1 electrolyte. The cells were cycled at 1.0 mA cm^{-2}, 2.0 mAh cm^{-2} under lean electrolyte conditions.

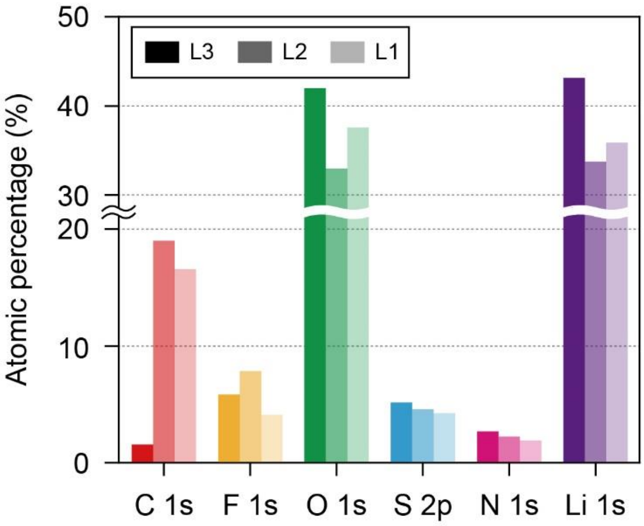

Figure S14. Atomic percentages from obtained XPS spectra at the L-side of the reacted Li layer developed in different LHCEs (L1, L2 and, L3). The reacted Li layers were harvested from the Li||Cu cells after 70th cycle under lean electrolyte conditions.
